# Investigation of the Role of Neurokinin-1 Receptor Inhibition Using Aprepitant in the Apoptotic Cell Death through PI3K/Akt/NF-*κ*B Signal Transduction Pathways in Colon Cancer Cells

**DOI:** 10.1155/2021/1383878

**Published:** 2021-08-02

**Authors:** Atefeh Ghahremanloo, Hossein Javid, Amir R. Afshari, Seyed Isaac Hashemy

**Affiliations:** ^1^Department of Clinical Biochemistry, Faculty of Medicine, Mashhad University of Medical Sciences, Mashhad, Iran; ^2^Student Research Committee, Faculty of Medicine, Mashhad University of Medical Sciences, Mashhad, Iran; ^3^Department of Medical Laboratory Sciences, Varastegan Institute for Medical Sciences, Mashhad, Iran; ^4^Department of Physiology and Pharmacology, Faculty of Medicine, North Khorasan University of Medical Sciences, Bojnurd, Iran; ^5^Surgical Oncology Research Center, Mashhad University of Medical Sciences, Mashhad, Iran

## Abstract

**Background:**

Colorectal cancer (CRC) is recognized as one of the most common malignancies with a high mortality rate worldwide, supporting the necessity for an effective novel antitumor drug to improve current therapy's effectiveness. Substance P (SP) is the essential member of the tachykinins (TKs) family, which binds to the specific receptors, known as neurokinin-1 receptor (NK1R), exerting its multiple influences such as tumor cell proliferation, angiogenesis, and metastasis. Aprepitant, as a specific NK1R antagonist, is suggested as a novel antitumor agent, promoting apoptotic processes in tumor cells; however, the exact antitumor mechanism of aprepitant on molecular signaling in CRC is not entirely known.

**Method:**

The resazurin assay was conducted to assess the cytotoxic effects of aprepitant on the viability of the CRC cell line (SW480). The level of reactive oxygen species (ROS) was measured after 24-hour treatment with SP and aprepitant. PI/annexin V-FITC staining was conducted to assess apoptosis. Also, the expression of NF-*κ*B antiapoptotic target genes and proapoptotic p53 target genes was measured by real-time- (RT-) PCR assay. Western blotting assay was performed to determine the expression of PI3k/AKT/NF-*κ*B proteins.

**Results:**

We found that aprepitant stimulates apoptotic cell death and attenuates the PI3K/Akt pathway and its downstream proapoptotic target gene, including NF-*κ*B in SW480 cells. Also, the obtained results from the quantitative RT-PCR assay showed that aprepitant could decrease the level of mRNA of NF-*κ*B antiapoptotic target genes.

**Conclusion:**

Towards this end, this study suggests that SP/NK1R system plays a vital role in the development of CRC, and pharmaceutical targeting of NK1R using aprepitant might be a promising treatment against CRC.

## 1. Introduction

Colorectal cancer (CRC) constitutes one of the commonly reported malignancies with limited treatment options and a high mortality rate [1]. In the recent years, there is a great deal of evidence that various agents and mechanisms are involved in the onset and progression of CRC [2–4]. In line with this, different molecular mutations have been shown in the advancement of CRC, including modifications in protooncogene KRAS, p53 tumor suppressor, and the transforming growth factor- (TGF-) *β* pathway [5]. Additionally, several observational researches have failed to indicate any therapeutic efficacy in the survival rate of CRC, despite evidence-based advances in treatment [6]. Toward this end, attention is focusing on developing new agents with potent antitumor properties to improve the treatment of CRC in the context of targeted therapies [7, 8].

Tachykinins' (TK) family consists of evolutionarily conserved neuropeptides that act as an immunomodulatory agent and regulate a diverse array of tumorigenesis processes in cells [9]. It is found that substance P (SP), hemokinin-1 (HK-1), neurokinin B (NKB), neurokinin A (NKA), and N-terminally extended forms of NKA, including neuropeptide *γ* (NP*γ*) and neuropeptide K (NPK), are the most important members of the family of TK neuropeptides in mammalian [10, 11]. The biological functions of these peptides are exerted by binding to specific receptors, entitled neurokinin 1 receptor (NK1R), NK2R, and NK3R [12]. As the leading central member of the TK family, SP has higher binding affinities to NK1R [7, 9]. It is well-known that SP is commonly presented in peripheral and central nervous systems, thereby playing an important role in regulating neurogenic inflammation and immune responses [13, 14].

Recently, an increasing number of cancer studies have been indicated that SP/NK1R system are overexpressed in many types of tumors, such as pancreatic, gastric, larynx, glioblastoma, colon cancer, and acute lymphoblastic leukemia cells [15–20]. Furthermore, recent studies have been demonstrated that SP/NK1R axis could lead to cancer progression, angiogenesis, and metastasis [21, 22]. Taking advantage of these characteristics, the concept of NK1R inhibition recently has received enormous attention as a novel therapeutic strategy in human cancer treatment.

Aprepitant (AP), as a highly specific NK1R antagonist, has already been approved by the Food and Drug Administration (FDA) for the prevention of chemotherapy-induced nausea and vomiting [23–25]. Besides, several pieces of evidence indicate that blocking of NK1R by AP resulted in a considerable reduction of mitogenic activity and inflammation process in some solid malignancies, including colon cancer [26–30]. Despite several studies on AP's antitumor role in different cancer cells, there is relatively little information about the intracellular signal transduction systems responsible for mediating AP's antitumor effects.

Taken together, to distinguish the critical role of the SP/NK1R signaling axis in CRC, our study has aimed to study the anticancer efficacy of AP in an associated CRC cell line (SW480).

## 2. Methods and Materials

### 2.1. Cells, Drugs, and Chemicals

SW480 cells were originally purchased from the Pasteur Institute of Iran (Tehran, Iran). RPMI 1640 medium (Gibco-BRL, Life technology, Paisley, Scotland) supplemented with 10% heat-inactivated fetal bovine serum (Gibco-BRL, Life technology, Paisley, Scotland) and 1% penicillin-streptomycin (Gibco-BRL, Life technology, Paisley, Scotland) were used for growing of cells. Cells were seeded in tissue culture flasks (Falcon, Heidelberg, Germany) and retained in a humidified incubator with 37°C and 5% CO2. For the administration of drugs, AP and SP were purchased from Sigma-Aldrich. The stock solution of these drugs was made in sterile dimethyl sulfoxide (DMSO) and culture medium, respectively. After that, they were divided into aliquots and stored at -80°C until use. DMSO concentration was added less than 0.1% of the total volume and adjusted as an applied solution.

### 2.2. Trypan Blue Exclusion Assay

To assess the inhibitory effects of AP on cell growth, cells were cultured at a density of 450 × 10^3^ cells/mL and treated with increasing concentration of AP (5, 10, 15, 20, 30, and 40 *μ*M) for 24 and 48 h. After that, 0.4% trypan blue and cell suspension were mixed in an equal proportion and incubated for 2 min at room temperature. Finally, the percentage of viable cells was evaluated as follows:

(1) $$\begin{array}{c} \%\text{cell viability}=\left(\frac{\text{viable cell count}}{\text{total cell count}}\right)\times 100. \end{array}$$

### 2.3. Growth Inhibition Studies

The cell metabolic activity of AP was assessed by resazurin-based cytotoxicity assay. Resazurin (7-hydroxy-3H-phenoxazin-3-one 10-oxide) is a nonfluorescent dye that can reduce to resorufin and dihydro-resorufin (highly fluorescent), which is directly dependent on the number of viable cells [31]. Briefly, 2.5 × 10^4^ cells were seeded in triplicates into 96-well plates (SPL Lifesciences, Pocheon, Korea). After the administration of cells with various concentrations of AP (5, 10, 15, 20, 30, and 40 *μ*M), the cells were incubated in a humidified incubator for 24 and 48 hours. The blue color of resazurin is altered to pink color resorufin and dihydro-resorufin by living cells. The fluorescent dye production was measured with a microplate fluorimeter under the excitation and emission wavelengths of 600 nm and 570 nm. The cytotoxic effect of AP was calculated by half-maximal inhibitory concentration (IC_50_) values measured with the dose-response curve in GraphPad Prism.

### 2.4. Measurement of ROS Activity

The level of intracellular ROS production was measured using 2′, 7′-dichlorodihydro fluorescein diacetate (DCFDA, Sigma, USA) following to the manufacturer's instructions. Briefly, after 24-hour incubation, cells were exposed to the DCFDA solution (20 *μ*M) for 30 min at 37°C. Afterward, cells were treated with SP (100, 400 nM) and AP (15 *μ*M) alone and in combination. Moreover, the tert-butyl hydroperoxide (TBHP) was used as a positive control group. Finally, the fluorescence intensity was measured at 495/529 (Excitation/Emission) in Perkin-Elmer Atomic Absorption Spectrophotometer.

### 2.5. Flow Cytometry Analysis of Apoptosis

Annexin V-FITC detection assay kit (Roche Applied Science, Germany) was used to detect apoptotic cell death as described previously [30]. Briefly, 35 × 10^4^ cells were seeded into 12 well plates, and the cells were collected after 24 h of treatment. Thereafter, cells were washed with PBS and added a total volume of 100 *μ*L of staining buffer, including annexin V-Flous solution (2 *μ*L/sample). Next, the plate was incubated for 30 min in the dark. Finally, the quantity of necrotic and apoptotic cells was measured by flow cytometer (BD Biosciences, San Jose, CA, USA). FITC-annexin V positive and PI negative were identified as early apoptosis, and annexin V positive, and PI-positive stained cells show late apoptosis.

### 2.6. RNA Extraction and Real-Time Quantitative PCR (qRT-PCR)

Total RNA extraction was realized from 65 × 10^4^ cells after treatment with SP (100 and 400 nM) and AP (15 *μ*M) and combination with each other using total RNA extraction mini kit (Yekta Tajhiz, Tehran, Iran), following the manufacturer's instructions. The RNA concentration was quantified by nanodrop spectrophotometer (NanoDrop 1000™, USA). Afterward, 1 *μ*g of RNA was reverse-transcribed to complementary DNA (cDNA) utilizing cDNA Synthesis Kit (Pars Toos, Tehran, Iran). Quantitative RT-PCR was performed with specific primers such as Bax, Bcl-2, survivin, and p53, purchased from Pishgaman (Pishgaman Co., Tehran, Iran) (Table 1). Also, glyceraldehyde-3-phosphate dehydrogenase (GAPDH) was used as a housekeeping gene and all amplifications were carried out in Roche real-time thermal cycler (Mannheim, Germany). The relative mRNA expression was calculated by the comparative Ct method (2^−ΔΔCt^).

### 2.7. Western Blot Analyses

The CRC cells were lysed with an ice-cold RIPA lysis buffer (250 mM Tris-HCl, pH 7.4, 0.5% sodium dodecyl sulfate (SDS), 5 mM EDTA, 750 mM NaCl, 5% Triton X-100) after exposing of sw480 cells with SP (400 nM) alone or with AP (10 *μ*M) [16]. Protein content was measured using bicinchoninic acid (BCA) protein assay kit (Thermo Scientific, Rockford, IL). The 30 *μ*g protein samples were loaded and separated by 10% sodium dodecyl sulfate-polyacrylamide gel electrophoresis (SDS-PAGE) and, subsequently, transferred onto nitrocellulose membrane. The membrane was incubated for 24 h at 4°C with specific antibodies against PI3K, Akt, *β*-Actin (1 : 1000; Cell Signaling, USA) and NF-*κ*B P65 (1 : 1000; Abcam, USA). Following two-hour incubation with anti-rabbit HRP-conjugated secondary antibodies (1 : 3000; Cell signaling, USA). Next, immune complexes on the membrane were detected by adding chemiluminescence detection kit (Thermo Scientific, Rockford, IL) according to the manufacturer's directions. Finally, band intensity was assessed with ImageJ software, and the ratio of proteins to actin expression was normalized.

### 2.8. Statistical Analysis

Experimental results are presented by mean ± standard deviation. The values were analyzed using ANOVA followed by Bonferroni's *t*-test through GraphPad Prism® 6.0 software (San Diego, CA, USA). Statistical significance was considered lower than 0.05 (*p* < 0.05).

## 3. Results

### 3.1. The Antiproliferative and Cytotoxic Effects of AP on Human CRC Cell Line SW480

**Figure 1:**
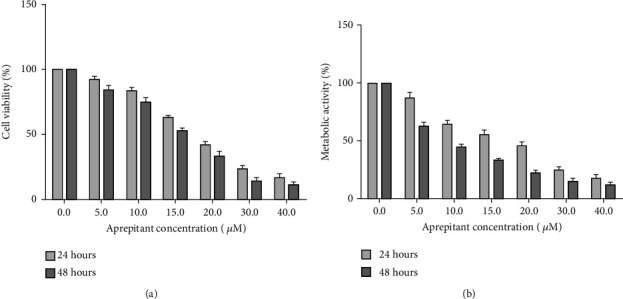
The inhibitory effect of AP in the proliferation and viability of SW480 cells. (a) AP has inhibitory activity on the viability of SW480 cells in a concentration and time-dependent based on trypan blue assay. (b) The inhibitory effects of AP on metabolic activity was assessed by resazurin assay after indicated times with doses escalation of AP. The IC50 value was evaluated about approximately 18 *μ*M after 24 h and 9 *μ*M for 48 h treatment. All data were shown as mean ± S.D of three independent experiments.

To investigate the cell growth inhibitory effects of AP, SW480 cells were exposed to increasing concentrations of AP (0-40 *μ*M) for 24 h and 48 h. On the basis of dose- and time-dependent manner, we found that elevating concentrations of AP (0-40 *μ*M) considerably decreased the cell survival and metabolic activity of SW480 cells (Figure 1). As illustrated in Figure 1(b), various concentrations of AP above the IC_50_ (approximately 18 *μ*M after 24 h and 9 *μ*M for 48 h treatment) metabolic activity of the SW480 cells is reduced. Additionally, according to the trypan blue exclusion assay (Figure1(a)), the cell viability is inhibited by various concentrations of AP (0-40 *μ*M) in a dose- and time-dependent manner. With respect to these results, the 10 *μ*M was chosen for experimental concentration. In 10 *μ*M of AP, cell viability and metabolic activity is approximately 65% as compared to untreated control.

### 3.2. Assessment of the Proapoptotic Effect of AP on Human CRC Cell Line SW480 Using Flow Cytometry

**Figure 2:**
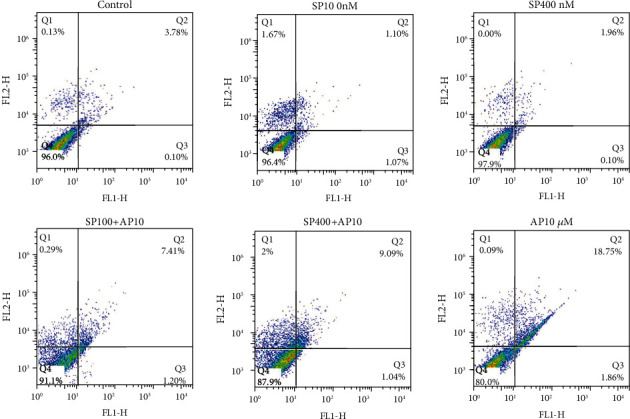
Proapoptotic effect of AP in SW480 cell lines. The percentages of annexin-V and annexin-V/PI double-positive in flow cytometry results implicate significantly increased apoptosis in the treated of SW480 cells with AP (10 *μ*M) as compared to untreated control cells.

To evaluate the efficacy of AP (10 *μ*M) as a single therapy or in combination with exogenous SP (100 and 400 nM) in programmed cell death, the cells were dual stained with annexin V-FITC and PI. Accordingly, our flow cytometry results (Figure 2) indicated that AP (10 *μ*M) as a single agent or in exposing to exogenous SP (100 and 400 nM) for 24 h could significantly increase the percentage of apoptotic and necrotic SW480 cells as compared to the control group (*p* < 0.05). Quantitatively, apoptotic and necrotic rates in the AP group were 20.7% while in the control groups were 4.01%. Taken together, these findings suggest that SP (100 and 400 nM) could attenuate apoptotic cell death in CRC cells (apoptotic and necrotic rates in the SP 100 nM group were 3.84% and in the SP 400 nM group were 2.06%).

### 3.3. The Negative Effect of AP on the PI3K/AKT Signaling Pathway in SW480 Cells

**Figure 3:**
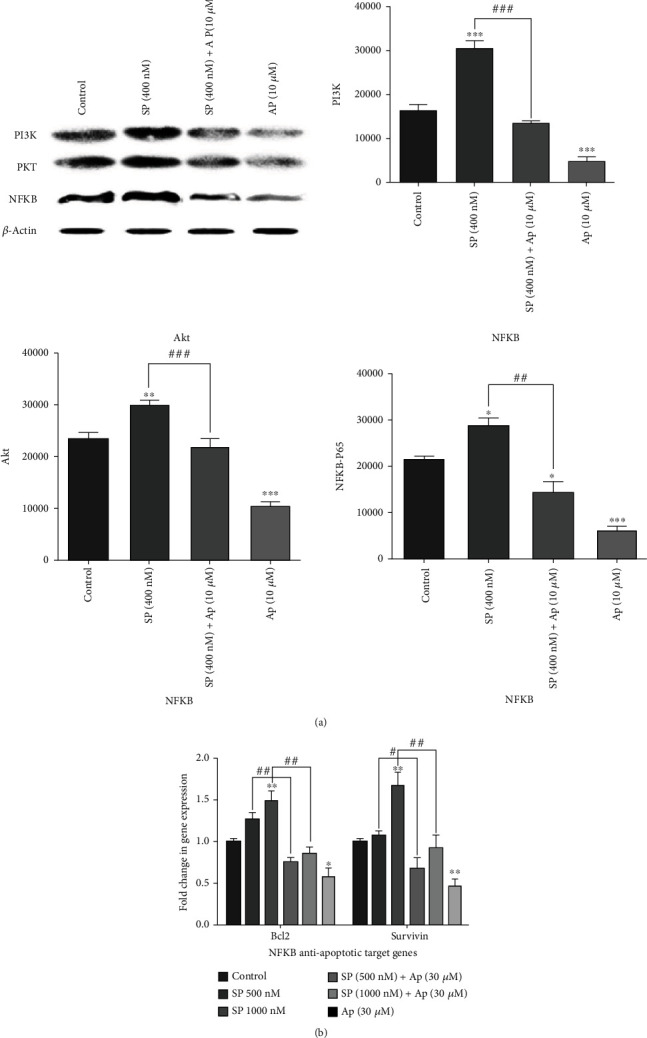
Abrogation of PI3K/Akt/NF-*κ*B signaling axis and downstream of antiapoptotic NF-*κ*B target genes by AP. (a) The cytoplasmic protein was extracted after cell treatment with AP (10 *μ*M) as a single agent or in combination with SP (400 nM), and Western blot assay was conducted using an Ab directed against NF-*κ*B P65, PI3k, Akt, and *β*-actin. The data were repeated at least two independent experiments. The intensity of bands was analyzed by the ImageJ software. (b) mRNA expression of NF-*κ*B antiapoptotic target genes was decreased by AP. GAPDH mRNA levels were used to normalized the levels of expression of all groups. All results were shown as a mean ± SD (*p* < 0.05).

Based on emerging evidence, the PI3K/Akt signaling axis is a potentially important treatment target in CRC [32]. Additionally, it has been found the SP/NK1R axis-induced activation of the PI3K and its downstream effectors such as total Akt molecules in various tumors [16, 30, 33, 34]. Towards this end, we assessed SP and AP's impact on the PI3K/Akt ratio in the SW480 cell line. According to the Figure 3(a), we found that the blocking of NK1R with AP alone (10 *μ*M) or in combination with exogenous SP (400 nM) after 24 h could attenuate the PI3K/Akt ratio. Also, these findings were associated with the negative effect of AP (10 *μ*M) on the PI3K/Akt signaling cascade.

### 3.4. Aprepitant Inhibits the NF-*κ*B Signaling Pathway and Attenuates Antiapoptotic Target Genes in CRC Cells

Recently, evidence has been shown that SP via binding to NK1R could enhance the NF-*κ*B signaling pathway activity and subsequently suppress cell apoptosis through targeting NF-*κ*B antiapoptotic target genes [8, 35]. The NF-*κ*B p65 subunit was assessed by Western blotting assay to increase the NF-*κ*B protein phosphorylation in response to SP (400 nM) and AP (10 *μ*M) in SW 480 cell line after 30 min. Furthermore, the mRNA expression of NF-*κ*B antiapoptotic target genes, such as Bcl-2 and survivin, was investigated by quantitative real-time PCR. We found that SP (400 nM) exerted an enhancer effect on the NF-*κ*B p65 subunit based on the obtained results. As shown in Figure 3(a), although the combination of SP (400 nM) with AP (10 *μ*M) decreased the NF-*κ*B protein expression, the AP (10 *μ*M) as a single agent has an additive anti-inflammatory effect compared to the untreated group. Moreover, the results of the mRNA expression level of NF-*κ*B downstream antiapoptotic target genes, such as surviving and Bcl-2, confirmed both the proinflammatory effect of SP and the anti-inflammatory effect of AP (10 *μ*M) in CRC cells (Figure 3(b)). Consistent with these findings, our results indicate that the SP and AP may have a critical regulatory role on the NF-*κ*B signaling pathway and downstream effectors.

### 3.5. Aprepitant Has No Significant Efficacy in the Enhancement of p53 and Proapoptotic p53 Target Gene Expression

**Figure 4:**
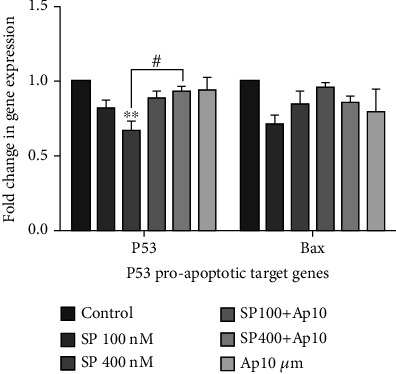
The effect of AP on the p53 and its proapoptotic target genes such as Bax. The expression levels of p53 and Bax were assessed with quantitative RT-PCR and were normalized with the expression level of GAPDH as indicated administration of AP has no significant efficacy on the expression levels of p53 and Bax (*p* < 0.05).

In addition to the NF-*κ*B axis, which is considered a crucial aspect in cancer cell apoptosis, the p53 and its proapoptotic target gene expression could induce apoptotic cell death in several human cancer cells [36–38]. Toward this end, we further evaluated the relative mRNA expression of p53 and downstream proapoptotic target genes such as Bax to investigate the SP/NK1R signaling activity in the SW480 cell line. As presented in Figure 4, our findings demonstrated that the mRNA expression of p53 and Bax was not significantly different among all groups compared to control. Taken together, these data showed that SP/NK1R system could induce a presumptive p53-independent apoptotic cell death in the SW480 cell line.

### 3.6. Aprepitant Decreased the Accumulation of Intracellular ROS in SW480 Cells

**Figure 5:**
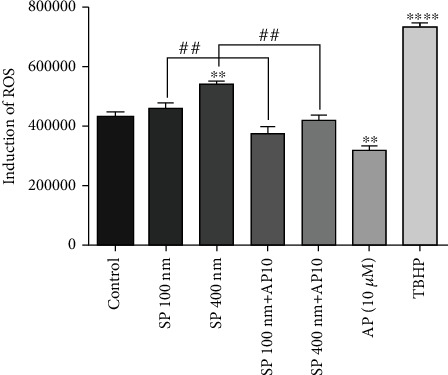
Reactive oxygen species formation is reduced following exposure to aprepitant. ROS formation was determined by DCFH-DA staining after administration of AP (10 *μ*M) alone or in combination with SP (100 and 400 nM). This figure confirms the inhibition efficacy of AP on intracellular ROS formation in the SW480 cell line.

Researchers have recently discovered that ROS generation promoting oxidative stress could mediate apoptotic cell death in various tumors [39–42]. Given these notions and the fact that apoptosis relies on the ROS level, we evaluated the influence of the SP/NK1R system on the ROS production level in SW480 cells. As illustrated in Figure 5, although the administration of SP (100 and 400 nM) could lead to increased intracellular ROS production after 24 h, the AP (10 *μ*M) with about 19% change in the AP10 *μ*M + sp100 nM group compared to the Sp100 nM group and about 22% change in the AP10 *μ*M + sp400 nM group compared to the Sp400 nM group or without about 25% change compared to the control group and the exogenous SP (100 and 400 nM) markedly attenuated the intracellular ROS level in CRC cells. Interestingly, these results confirmed the previous observations in NF-*κ*B downstream antiapoptotic target genes. Taken together, these data supported the antiapoptotic effects of AP through decreased ROS production in CRC cells.

## 4. Discussion

Since SP interaction with NK1R is involved in the development and progression of multiple cancers, an increasing number of investigations focus on blocking human NK1R by various antagonists [6, 27]. Additionally, it is known that the expression of NK1R increases on the surface of tumor cells, such as CRC [17–20]. In the context of cancer treatment, the antitumor influence of AP, as an oral NK1R antagonist, opens up a new avenue to cancer treatment, including melanoma, hepatoblastoma, pancreas, lung, and breast malignancy [21, 28, 43–46]. In line with this, we evaluated the antitumor effect of AP on the molecular signaling in CRC. For this purpose, we found that AP has concentration-dependent antiproliferative and cytotoxic activity on human CRC cell line SW480. As illustrated in Figure1, cell viability and metabolic activity of cells after treatment with 10 *μ*M of AP are approximately 65% as compared to untreated control. We also determined AP-induced apoptotic processes in the SW480 cell line using flow cytometry and found promising results in the treated group with AP. As shown in Figure 2, apoptotic and necrotic cells in the AP group were 20.7% while in the control group were 4.01%. Simply putting analysis of flow cytometry data implied that the blockage of NK1R leads to inhibit the proliferation of CRC cells and stimulate apoptosis in SW480 cells. Similarly, a recent study reported promising results in inducing apoptosis of esophageal squamous cell carcinoma cells after treatment with AP. They found that treatment of esophageal squamous cell carcinoma with AP (15 *μ*M) can impose approximately 83.5% of cells to apoptosis and necrosis, while the apoptotic and necrotic cells were 10.23% in control group [30]. Furthermore, Bayati et al. demonstrated that blockade of NK1R on the surface of acute lymphoblastic leukemia cells with several concentrations of AP (10, 20, and 30 *μ*M) leads to an increase in the percentage of apoptotic and necrotic cells. In this regard, their results show that AP 30 *μ*M induces 59% of cells to apoptosis, while the percentage of apoptotic cells after treatment with AP 20 *μ*M and AP 10 *μ*M was about 25% and 29%, respectively [16].

Additionally, our data demonstrated that the blockage of NK1R with AP (10 *μ*M) could decrease the PI3K/Akt ratio, supporting the findings of apoptosis results. Our results agreed with the data obtained from the recent studies that reported inhibition of NK1R cause to suppress the PI3K/AKT signaling axis and resulted in apoptotic cell death [16, 30, 34]. In line with this, it is known that the PI3K/AKT regulates various transcriptional factors such as NF-*κ*B, leading to imposing several cellular react including apoptosis cell death, invasion, and inflammation [47, 48]. Toward this end, we tried to show that whether AP could stimulate apoptotic cell death by modifying the PI3K/Akt/NF-*κ*B signaling pathway (Figures 3(a) and 3(b)). Our results demonstrated that the treated group with AP (10 *μ*M) decreases both expression of NF-*κ*B p65 protein and mRNA expression of NF-*κ*B antiapoptotic target genes such as survivin and Bcl-2 (Figures 3(a) and 3(b)). To further investigate apoptotic mechanisms by AP, recent evidence has been found that inducing apoptosis by AP is associated with increased p53 in several tumor cells [16, 35, 36]. In support of this idea, it is well found that the activation of PI3K/Akt is correlated with p53 pathways [16]. In contrast to these results, we found that the AP (10 *μ*M) could not increase the mRNA expression level of p53 and, subsequently, the mRNA level of proapoptotic p53 target genes such as Bax (Figure 4). Moreover, in accordance with our data, Javid et al. reported that AP induce program cell death through a p53-independent apoptotic pathway in esophageal squamous cell carcinoma [30]. In harmony with AP-inhibited PI3K/Akt/NF-*κ*B, these data supported that AP might apply its therapeutic effects through a p53-independent apoptotic axis in SW480 cells.

Additionally, it is well established that mitochondrial ROS elevation plays a vital role in apoptosis induction [49, 50]. Furthermore, intracellular production of ROS is associated with activation of the NF-*κ*B signaling pathway and the suppression of apoptotic cells death, survival, and critical role in tumorigenesis of cancer cells [51, 52]. Given this, as illustrated in Figure 5, our data indicated that AP could reduce about 25% change ROS production compared to the control group based on the NF-*κ*B axis regulated in SW480 cells.

## 5. Conclusions

**Figure 6:**
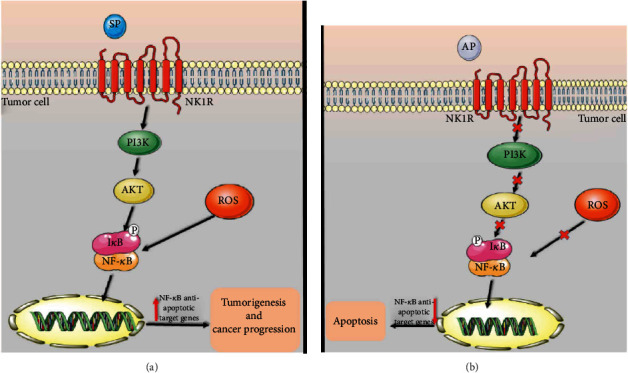
Schematic representation of the possible mechanism of the SP/NK1R system and aprepitant in CRC-derived SW480 cells. (a) A model of signaling of the SP/NK1R system in the absence of aprepitant. (b) A model of signaling of the SP/NK1R system in exposure to aprepitant.

Overall, our research study revealed that blockage of NK1R with the specific antagonist, AP, has displayed anticancer efficacy against the SW480 human CRC cell line by abrogating PI3K/Akt/NF-*κ*B axis (Figure 6). Additionally, according to the pharmacologic safety of AP against chemotherapy-induced nausea and vomiting as a positive characteristic, our outputs suggest that AP can play an essential therapeutic against CRC as a single therapy or in combination with other typical anticancer therapeutics. However, despite numerous studies on the antitumor activity of AP against various tumor cells, more researches are required to further elucidate the underlying functional mechanisms of AP in multiple cancers.

## Figures and Tables

**Table 1:** Nucleotide sequences of the primers used for real-time RT-PCR.

Gene	Source	Forward primer (5′-3′)	Reverse primer (5′-3′)
GAPDH	Human	5′-ACAACTTTGGTATCGTGGAAGG-3′	5′-GCCATCACGCCACAGTTTC-3′
Bax	Human	5′-CGAGAGGTCTTTTTCCGAGTG-3′	5′-GTGGGCGTCCCAAAGTAGG-3′
Bcl2	Human	5′-CGGTGGGGTCATGTGTGTG-3′	5′-CGGTTCAGGTACTCAGTCATCC-3′
Survivin	Human	5′-CCAGATGACGACCCCATAGAG-3′	5′-TTGTTGGTTTCCTTTGCAATTTT-3′
P53	Human	5′-GAGGTTGGCTCTGACTGTACC-3′	5′-TCCGTCCCAGTAGATTACCAC-3′

## Data Availability

Data are available upon request.
